# Solid Pseudo-Papillary Tumor of the Pancreas in a 10 Year Old Girl

**Published:** 2015-07-20

**Authors:** K Goudarzipour, A Jenabzadeh, H Mirzaei Ilali, B Behnam, H Tavassol

**Affiliations:** 1 **Pediatric Congenital Hematologic Disorders Research Center, Shahid Beheshti University of Medical Sciences, Tehran, Iran.**; 2 **Functional Neurosurgery Research Center, ShohadaTajrish Hospital, Shahid Beheshti University of Medical Sciences, Tehran, Iran.**

**Keywords:** Solid pseudo-papillary tumor of the pancreas

## Abstract

**Background**

Solid pseudo-papillary tumor of the pancreas (SPTP) is a rare disease with a low malignant potential. Though it shows low malignant potential 10% to 15% of the cases show aggressive behavior with metastatic involvement of the liver. The symptoms include abdominal discomfort and abdominal pain. It is very rare in early years of age. This is the case of a 10 year old girl with abdominal pain and her evaluation revealed solid pseudo papillary tumor of pancreas. In family history, her grandmother died because of pancreas cancer. The mass was excised and in her 6-month follow up she didn’t have any problems. This case is presented to point out physicians that more attention to pseudo- papillary tumor can bring us significant improvement in the diagnosis of this pathology, though pseudo- papillary tumor is a rare pathologic condition in children.

## Introduction

Solid pseudo-papillary tumor of the pancreas (SPTP) represents less than 10% of cystic neoplasm of the pancreas. SPTP is a rare neoplasm that affects mainly young females. Though it shows low malignant potential, 10% to 15% of the cases show aggressive behavior with metastatic involvement of the liver. The overall five year survival is 97% even in the presence of disseminated disease [1].

The sign and symptom of SPTP is related to mass effect and consists of abdominal pain and abdominal discomfort. Physical examination shows a palpable and significant mass when the symptoms present. These tumors usually have large size with average diameter of 8-10 cm. serologic tumor marker and laboratory tests are in normal ranges. CT scan is an imaging technique of choice for diagnosis and shows a well-defined large solid-cystic mass. Prognosis is excellent and surgical resection can result in complete cure.

## Case Report

A 10 years old girl was admitted to the hospital complaining of peri umblical pain. She had suffered from diarrhea and vomiting from one month ago that disappeared after few days and followed by these symptoms: squeezing and abdominal pain. There were no systemic symptoms such as weight loss or etc before admission. Her grandmother died from pancreatic cancer but there wasn't any information about type of her tumor.

**Figure 1:**
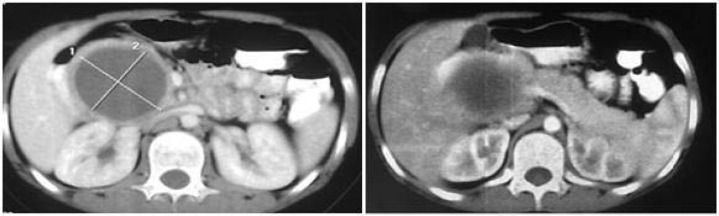
Axial view of abdomenal CT scan with a retroperitoneal mass with measuring 60*58 mm in size compressed liver and right kidney vessels

Sonography and CT scan showed a retroperitoneal mass with measuring 60*58 mm in size compressed liver and right kidney vessels (Fig 1).

**Figure 2:**
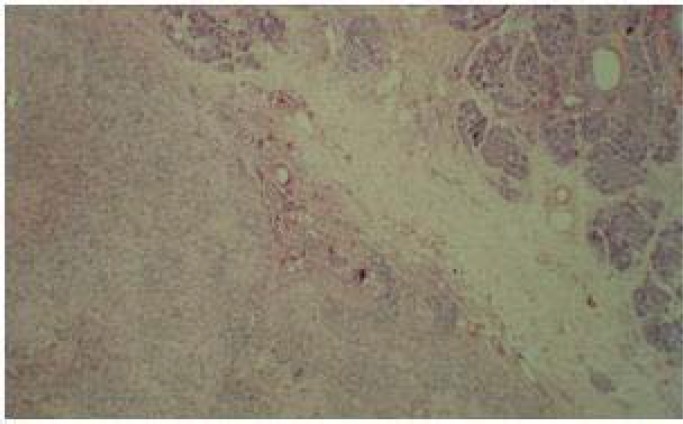
Pathology of Solid Pseudo-Papillary Tumor of the Pancreas

Tumor markers like CEA and CA19-9 were normal, however CA125 had increased. Other lab tests such as CBC and electrolytes were also normal. The Lowercase underwent surgical laparatomy and after mass excision, the pathologist reported a pseudo-papillary tumor with infiltration to ampulla of vater (Fig 2).

Patient was discharged with good condition 4 days after surgery. In her follow-up, after 6 months, her condition was good and level of CA125 had returned to normal range.

## Discussion

Solidpseudo-papillary tumor (SPT) is a rare pancreatic neoplasm and it has low potential for malignancy. It is also a rare condition in males and children and occurs mostly in young women. The most common presentations of SPT in children are abdominal mass and abdominal pain.[2]SPT usually can be revealed with nonspecific clinical presentations. The most common symptom is abdominal pain that can be followed by an enlarged abdominal mass [3].

Pancreatic tumors are classified in 3 following categories: consist of exocrine, endocrine, and mesangial tumors. Papillary tumors account for 1% to 2% of exocrine tumors of pancreas. According to WHO classification, SPT with clear criteria of malignancy (i.e. vascular and nerve sheath invasion, lymph node and liver metastasis) are designated as solid pseudo papillary carcinomas. 

It usually affects young women between second and third decades of their life. Mostly, SPTs manifested as benign tumors may exhibit malignant behavior with metastatic involvement of adjacent structures. Patient with SPT of pancreas may suffer from uncharacteristic abdominal pain, enlarged abdominal mass which compressed adjacent viscera. It may be asymptomatic that is diagnosed incidentally on ultrasonography or CT scan .The etiology of these tumors is unknown. Size of tumors can reach up to 18cm but rarely have metastatic presentation. [4]CT scan is the most widely used imaging technique in the diagnosis. It is capable of showing the characteristic but non-specific pattern including presence of large encapsulated mass in contact with pancreatic tissue with peripheral contrast enhancement, solid and cystic areas. Occasionally, calcification and bleeding signs may be seen inside the tumor.[5,6,7] it is Supposed that surgical excision of tumor is an appropriate therapeutic strategy for these patients. A retrospective study from 1992 to 2002 has reported 14 cases with pseudo papillary tumor of pancreas that all of them were female in the age group of 13-45 years.[8] The importance of this case report are the young age and family history of pancreatic cancer which resulted to her grandmother’s death . The patient underwent only surgical resection and in 6 month follow-up she had no problem.

## Conclusion

Abdominal pain and abdominal mass are common presentations of SPTs. These tumors occur mostly in young females and they have low potential for malignancy. They are respectable in most cases and associated with well prognosis.

SPTs are not only inactive and slow in comparison to other pancreatic malignancies, though they can also be presented with invasive metastatic tumors.

Metastasis has been seen in only 10%–15% of patients who have advanced stages of disease at the time of diagnosis.
